# Medical Outcomes in Women Who Became Pregnant after Vaccination with a Virus-Like Particle Experimental Vaccine against Influenza A (H1N1) 2009 Virus Tested during 2009 Pandemic Outbreak

**DOI:** 10.3390/v11090868

**Published:** 2019-09-17

**Authors:** Arturo Cérbulo-Vázquez, Lourdes Arriaga-Pizano, Gabriela Cruz-Cureño, Ilka Boscó-Gárate, Eduardo Ferat-Osorio, Rodolfo Pastelin-Palacios, Ricardo Figueroa-Damian, Denisse Castro-Eguiluz, Javier Mancilla-Ramirez, Armando Isibasi, Constantino López-Macías

**Affiliations:** 1 Facultad de Medicina, Plan de Estudios Combinados en Medicina (MD, PhD Program), Universidad Nacional Autónoma de México, Mexico City CP 04510, Mexico; cerbulo@unam.mx; 2 Unidad de Investigación Médica en Inmunoquímica, Hospital de Especialidades del Centro Médico Nacional Siglo XXI, Instituto Mexicano del Seguro Social (IMSS), Mexico City CP 06720, Mexico; landapi@hotmail.com (L.A.-P.); ibosco45@hotmail.com (I.B.-G.); isibasi@prodigy.net.mx (A.I.); 3 Escuela Nacional de Ciencias Biológicas, Programa de Inmunología, Instituto Politécnico Nacional, Mexico City CP 11340, Mexico; gabrielacruz30@gmail.com; 4 Servicio de Cirugía Gastrointestinal, Unidad Médica de Alta Especialidad, Hospital de Especialidades Dr Bernardo Sepúlveda Gutiérrez, Centro Médico Nacional Siglo XXI, Instituto Mexicano del Seguro Social (IMSS), Mexico City CP 06720, Mexico; eduardoferat@prodigy.net.mx; 5 Departamento de Biología, Facultad de Química, Universidad Nacional Autónoma de México, Mexico City CP 04510, Mexico; rodolfop@unam.mx; 6 Departamento de Infectología, Instituto Nacional de Perinatología, Mexico City CP 11000, Mexico; rfd6102@yahoo.com.mx; 7 Consejo Nacional de Ciencia y Tecnología (CONACYT)- Departamento de Investigación Clínica, Instituto Nacional de Cancerología, Mexico City CP 14080, Mexico; angeldenisse@gmail.com; 8 Escuela Superior de Medicina, Instituto Politécnico Nacional; Hospital de la Mujer, Secretaria de Sauld, Mexico City CP 11340, Mexico; javiermancilla@hotmail.com; 9 Visiting Professor of Immunology, Nuffield Department of Medicine, University of Oxford, Oxford OX3 7LF, UK; 10 Mexican Translational Immunology Research Group, Federation of Clinical Immunology Societies Centers of Excellence, National Autonomous University of Mexico, Mexico City 04510, Mexico

**Keywords:** virus-like particle, influenza A(H1N1)pdm09, vaccination, pregnant women, antibody titers

## Abstract

The clinical effects and immunological response to the influenza vaccine in women who later become pregnant remain to be thoroughly studied. Here, we report the medical outcomes of 40 women volunteers who became pregnant after vaccination with an experimental virus-like particle (VLP) vaccine against pandemic influenza A(H1N1)2009 (influenza A(H1N1)pdm09) and their infants. When included in the VLP vaccine trial, none of the women were pregnant and were randomly assigned to one of the following groups: (1) placebo, (2) 15 μg dose of VLP vaccine, or (3) 45 μg dose of VLP vaccine. These 40 women reported becoming pregnant during the follow-up phase after receiving the placebo or VLP vaccine. Women were monitored throughout pregnancy and their infants were monitored until one year after birth. Antibody titers against VLP were measured in the mothers and infants at delivery and at six months and one year after birth. The incidence of preeclampsia, fetal death, preterm delivery, and premature rupture of membranes was similar among groups. All vaccinated women and their infants elicited antibody titers (≥1:40). Women vaccinated prior to pregnancy had no adverse events that were different from the nonvaccinated population. Even though this study is limited by the sample size, the results suggest that the anti-influenza A(H1N1)pdm09 VLP experimental vaccine applied before pregnancy is safe for both mothers and their infants.

## 1. Introduction

Vaccination against pandemic influenza A is crucial for limiting the incidence and severity of infection in the general population [1,2]. Influenza A infection in pregnant women has been associated with higher admission rates to intensive care units and severe disease compared with nonpregnant women [3]. Studies in animal models show that the seasonal influenza virus infection in pregnant mice initiates a powerful pro-abortive mechanism and adverse outcomes in fetal health [4], highlighting the importance of vaccination in protecting both pregnant women and their infants to whom immunity may also be passively transferred [5,6].

In 2009, our group conducted a phase 2 clinical trial following Good Clinical Practices according to the International Conference of Harmonization (ICH-GCP) to evaluate the safety and immunogenicity of a virus-like particle (VLP) vaccine against influenza A(H1N1)pdm09. That study was a randomized, placebo-controlled, double-blind clinical trial that included 4555 male and female volunteers. Healthy nonpregnant women (confirmed by a negative pregnancy test) were included in the study [7,8], and by written informed consent, women were requested to avoid pregnancy for the duration of the trial. The use of a contraceptive method was instructed and provided to female participants upon request; however, some of the volunteers became pregnant in the postvaccination follow-up period. As indicated by our institutional Ethics Committee recommendations and local regulations, the pregnant women and their infants remained under the intention to treat protocol, so they were closely monitored. The medical outcomes and antibody response to the vaccine in both the women and their infants were registered. Here, we report, in a nested cohort study, the results of the surveillance of the 40 pregnant women who agreed to take part and provided their informed consent. 

## 2. Materials and Methods 

While conducting an ICH-GCP phase 2 clinical trial using a VLP vaccine against pandemic influenza A at the Mexican Institute of Social Security (IMSS, Mexico City, Mexico), 4555 male and nonpregnant female (confirmed by a negative pregnancy test) volunteers were included. Despite the use of contraceptive methods—as stated by the participants—40 women reported pregnancy in the postvaccination period (Table 1). We reported these events to the IMSS Ethics Committee and Comisión Federal para la Prevención de Riesgos Sanitarios (COFEPRIS) (the Mexican equivalent of the US Food and Drug Administration), and they both recommended that the pregnant women and their infants should remain under medical surveillance and be closely monitored according to local regulations.

**Figure 1:**
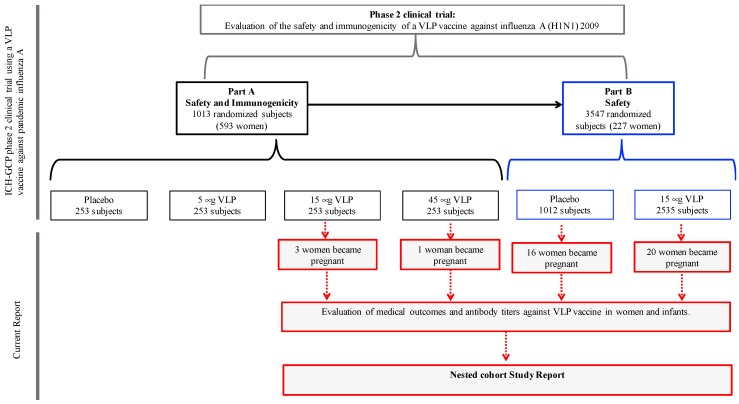
Flow and details of the subjects in the trial. A total of 820 women volunteers participated in the phase 2 clinical trial to evaluate the safety and immunogenicity (part A) and safety (part B) of the VLP vaccine against influenza A(H1N1)pdm09 [6]. After vaccination, 40 women became pregnant—16 from the placebo group, 23 from the 15 μg VLP vaccine dose, and 1 from the 45 μg VLP vaccine dose. All these volunteers were provided with medical surveillance and close monitoring; clinical outcomes and VLP vaccine specific antibody titers in both mothers and their infants were evaluated. Both the Mexican Institute of Social Security and the National Institute of Perinatology Ethic Committees approved the study (IMSS:R-2011-785-040, INPer:212250-06181).

The design of this study was a nested cohort study that included the 40 women who became pregnant after the influenza A(H1N1)pdm09 virus vaccination and their infants (Figure 1).

### 2.1. Participant Characteristics

The 40 women who became pregnant after vaccination were recruited from the VLP vaccine clinical trial groups—16 (40%) pregnant women from the placebo group, 23 (57.5%) from the 15 μg dose of VLP vaccine group, and 1 (2.5%) woman from the 45 μg dose of VLP vaccine group (Figure 1). None of the women had documented an infection with pandemic influenza A (H1N1) 2009 or a vaccination against seasonal or pandemic influenza A (other than the experimental vaccine), and none of them reported a medical history of chronic diseases.

The pregnant women were monitored medically until delivery, following the standard protocol for medical care in Mexico [9]. Both mothers and infants remained under medical surveillance and safety follow-up at 3, 6, and 12 months after delivery. Any adverse medical or perinatal event experienced by the mothers or infants was recorded in detail. The newborn surveillance included anthropometry, gestational age at birth, nutritional status, and congenital disease.

The IMSS and the National Institute of Perinatology Ethic Committees approved the study (IMSS:R-2011-785-040, INPer:212250-06181). All participants signed a written informed consent for the study.

### 2.2. Sample Collection 

Whole blood samples (5 mL) from the pregnant women or umbilical cord blood (10 mL) were collected at birth. At 3, 6, and 12 months of age, 5 mL of peripheral blood was collected from the mothers and 1 mL from the infants. Serum was obtained from the blood samples by centrifugation, and the aliquots were stored at −20 °C until analysis.

### 2.3. Hemagglutination Inhibition (HAI) Test

The serum samples were tested in triplicate. Assays were conducted as previously described [10,11]. The aliquots of each serum sample were treated using the receptor-destroying enzyme. The samples were diluted (1:2) into V-bottom 96-well microtiter plates. Eight units of 50 μL of hemagglutinin (HA) were added to each well, plated, and incubated for 30 min at room temperature, followed by the addition of in-house, freshly prepared turkey erythrocytes (1% in phosphate-buffered saline). The plates were mixed by agitation, covered, and allowed to set for 30 min at 25 °C. The HAI titer was determined by the reciprocal of the last dilution of nonagglutinated red blood cells. Samples with HAI titers of ≥1:40 were considered to have a positive antibody response to the influenza A(H1N1)pdm09 virus.

### 2.4. Statistical Analysis 

Descriptive statistics were used for the characterization of the patients studied. We compared the placebo and VLP 15 μg dose. The statistical analysis was performed using StataCorp software (2011 Stata Statistical Software—Release 12, College Station, TX: StataCorp LP). Descriptive statistics were used to characterize the categorical variables, expressed as a percentage. A bivariate analysis was performed for comparison among groups, using Fisher’s exact test or chi-square test; *p* < 0.05 was considered statistically significant.

## 3. Results

### 3.1. Medical Outcomes in Women Who Became Pregnant after Vaccination with VLP Vaccine against Influenza A(H1N1)pdm09 Virus and Their Infants

Table 1 describes the characteristics of the women studied. All volunteers included in the study were residents of the metropolitan area of Mexico City and were rightful claimants of full cover medical attention provided by the public health system; all were homemakers, literate, and none reported a medical history of chronic diseases (Table 1). The mean age was 26.1 ± 5.4 years (see Table 1 for the differences in characteristics among the placebo and 15 μg VLP groups). The mean time to become pregnant after vaccination was 130 ± 115 days; the details on the elapsed time from vaccination to pregnancy are described in Table 1.

The gestational age of the newborns and the frequency of the delivery type were similar between the placebo and 15 μg VLP vaccine groups (Table 2). A similar average birth weight of newborns was observed between the placebo group (2878 ± 554 g) and the 15 µg VLP vaccine group (3081 ± 398 g; *p* > 0.05). The woman from the 45 µg VLP vaccine group delivered via cesarean section at 38 weeks of gestational age, the newborn had a birth weight of 3000 g and an Apgar score typical for a healthy newborn (8/9, 1 min and 5 min).

None of the pregnant women from the placebo group were diagnosed with preeclampsia, fetal death, premature rupture of membranes, oligohydramnios, or gestational hypertension (Table 3). Two of the women from the VLP 15 µg group received a second immunization (28 days after the first dose); no obstetric complications or adverse events developed in these two women. Six (26%) of the women in the 15 μg VLP group experienced adverse events. These events were preeclampsia (*n* = 2), fetal death (*n* = 1), premature rupture of membranes (*n* = 1), oligohydramnios (*n* = 1), and gestational hypertension (*n* = 1). Table 3 describes the obstetric complications for both the placebo and 15 µg VLP groups; the epidemiological reference of obstetric adverse events reported for the Mexican population is also provided [12]. In all cases, the frequency of the above-mentioned events was not significantly different among the placebo and 15 μg VLP groups (Table 3). The patient who received the 45 µg VLP vaccine did not have any pathology during her pregnancy. In the placebo group, one patient had a preterm birth delivered by cesarean section at 36 weeks, whereas 2 out of 23 (8.7%) women in the 15 μg VLP vaccine group had a cesarean preterm birth at 35 and 36 weeks of gestational age. The woman in the 45 μg VLP vaccine group delivered at term gestational age.

We evaluated 32 infants during the first year of life. Seven abandoned the study during follow-up, and in one case, consent was withdrawn. One infant (6.2%) from the placebo group was diagnosed with pneumonia during the surveillance period. In the 15 μg VLP group, one infant (4.3%) was diagnosed with a hydrocele, one infant (4.3%) was diagnosed with an umbilical hernia, and one infant (4.3%) was diagnosed with gastroesophageal reflux and bacterial pneumonia during medical surveillance (Table 4). Low birth weight and fetal distress were not statistically different between the placebo and 15 μg VLP vaccine groups (Table 4). The infant exposed to the 45 µg VLP dose did not present any pathology.

### 3.2. Vaccination Elicited Humoral Response in Pregnant Women and Infants

**Figure 2:**
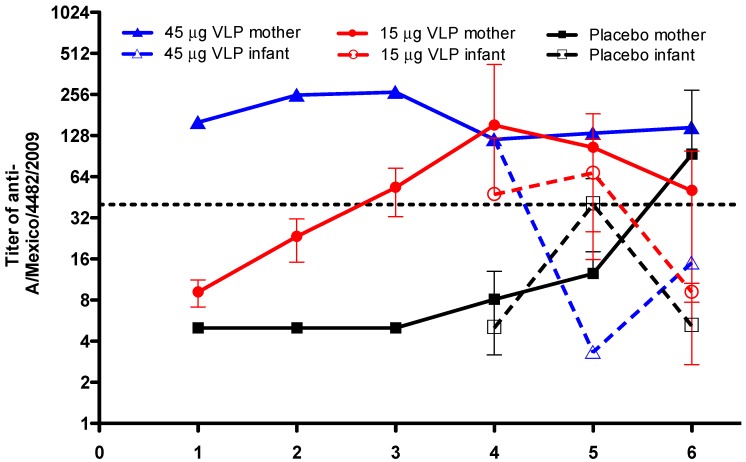
VLP vaccination induces humoral response against the anti-A/Mexico/4482/2009 virus in mothers and infants. Antibody titers in women volunteers vaccinated with 15 μg VLP (*n* = 23), 45 μg VLP (*n* = 1), or placebo (*n* = 16). D0—day of vaccination (first dose); D22—day 22 after vaccination (also, two women in the 15 μg group received a second dose); D36—day 36 after vaccination; birth—day of delivery of the newborns; 6 months—6 months after birth; and 12 months—12 months after birth. Closed symbols and continuous lines show antibody titers in women. Open symbols and dashed lines show antibody titers in infants. Mean ± SD.

Figure 2 shows the titer of antibodies against pandemic influenza in the peripheral blood of women who were vaccinated before becoming pregnant. Women in the placebo group showed low levels of antibodies against pandemic influenza at the beginning of the trial; however, these levels increased from delivery up to one year after delivery, and the titer reached high levels (>1:40) at 12 months after delivery. The titer was less than 1:40 in the placebo infants, reaching the highest levels at six months after birth. In the 15 μg VLP vaccine group, the antibody titer in women increased as early as 20 days after vaccination and reached high levels at 36 days after the vaccination, whereas at delivery, the titer was higher than 1:80 and then decreased to 1:40 at 12 months after delivery. The titer in the infants from the 15 μg VLP group was 1:40 at the moment of birth and remained elevated at six months of age; however, the titer fell below 1:40 at 12 months after birth (Figure 2). In the woman from the 45 μg VLP vaccine group, the initial titer was 1:160 and remained protective from the moment of vaccination until one year after delivery; the infant from this group showed a similar titer as the mother at birth, but it diminished at six months of age and remained below 1:40 at 12 months after birth (Figure 2). 

**Figure 3:**
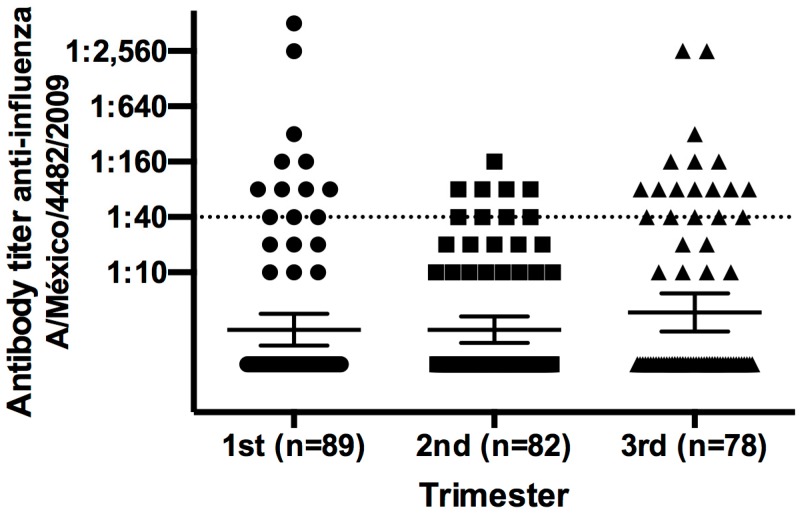
Protective antibody titer against pandemic influenza in healthy Mexican pregnant women. We collected 249 peripheral blood samples from healthy pregnant women who did not participate in the vaccination clinical trial—12 out of 89 women (13%) in the first trimester, 9 out of 82 (12%) in the second trimester, and 18 out of 78 (30%) in the third trimester had an antibody titer ≥1:40. Mean ± SD.

To understand the elevated antibody titers observed in women and infants from the placebo group, we determined the antibody titers against pandemic influenza in 249 pregnant women who did not participate in the vaccination trial (Figure 3). About 30% of Mexican women who became pregnant in the period from July 2009 to August 2010 expressed natural immunity and antibody titers of 1:40 against pandemic influenza, which is similar to the antibody titers described for the placebo group.

## 4. Discussion

Here we have reported the results of a nested cohort study that evaluated the potential effect of the anti-influenza A(H1N1)pdm09 VLP experimental vaccine in women who later became pregnant on medical outcomes as well as on the antibody response of the women and their infants [7]. Tavares et al. [13] reported similar results in their study of the effect of a pandemic vaccine against influenza A (H1N1) in pregnant women using an AS03-adjuvanted split virion H1N1 (2009) pandemic influenza vaccine. They did not observe an increase in the frequency of obstetric complications, infection, or teratogenicity. Tamma and Muñoz [14,15] reported no adverse effects in the infants of mothers vaccinated using an inactivated virus. 

We monitored 40 women from early gestation to delivery and 32 infants during the first year of life. Although this study may lack statistical power due to the small sample size, our results showed that most women in this cohort reached term gestational age (37–42 weeks); this could suggest that the VLP vaccination does not result in a higher risk of preterm birth than occurs in the nonvaccinated population, as the average gestational age was similar to the Mexican national epidemiological data of term newborns [16].

We did not observe an increase in the rates of maternal obstetric complications or infection. The incidences of preeclampsia, fetal death, and premature rupture of membranes were not significantly higher in the VLP-vaccinated women compared to nonvaccinated women, suggesting that the VLP vaccine is safe in the pregnant population even under conditions of influenza outbreak. A prospective and larger study should be conducted to ascertain the safety of the VLP vaccine in the pregnant population.

The rates of congenital disease, fetal death, preterm delivery, and low birth weight in the neonates of VLP-vaccinated women were similar to that of the nonvaccinated population, suggesting that the VLP vaccine does not result in a higher risk to the fetus. In the VLP vaccine group, there were three cases of low birth weight and two cases of fetal distress, even though no statistical significance was reached, this finding may be clinically relevant and considered for further studies. Pediatric monitoring showed no negative effects on the exposed infants; cases of hydroceles, umbilical hernias, and gastroesophageal reflux are common in Mexican newborn infants, and it was not possible to establish a causal factor between the application of the influenza vaccine prior to pregnancy and the occurrence of these complications. However, the effect of the VLP vaccine applied in pregnant women must be studied to provide confidence in the safety of this type of vaccination.

Vaccination with an inactivated or attenuated virus for influenza A has some disadvantages, such as the risk of anaphylactic reactions and requirements for annual boosters or high doses to reach protection [17,18,19,20]. We observed specific antibody titers to A/Mexico/4482/2009 in pregnant women in the placebo group; these antibodies may be passively transferred to the fetus. As we observed, these antibody titers are maintained up to 12 months after birth. About 30% of Mexican women who became pregnant during the period of the pandemic showed elevated antibody titers; accordingly, our data suggest that some women in the placebo group may have been naturally infected by the influenza virus and developed an antibody response that could be transferred to the fetus. One dose of 15 µg VLP vaccine was shown to elicit a transitory but protective titer in the perinatal period, which was maintained 12 months after delivery. A similar titer was detected in infants during the newborn period up to six months after birth, suggesting that one dose of 15 μg VLP is sufficient to elicit an antibody response. Considering 60% of these infants were breastfed throughout the first year of life, it is possible that antibodies may have been passively transferred during this period; thus, the 15 µg VLP dose may be considered sufficient to support passive immunity in the fetus and infant. Finally, the high titer that we found in the woman who was exposed to one dose of 45 µg VLP vaccine suggests an asymptomatic infection with the pandemic influenza virus, and this condition may have interfered with the response to the 45 µg VLP vaccine. However, the titer remained elevated and the antibodies were passively transferred to the neonate at the moment of delivery but were not sustained at six months. Taken together, our data suggest that 15 µg may be an adequate dose of VLP vaccine to elicit an adequate antibody titer in both mother and infant. Another clinical trial is necessary to demonstrate if the 15 µg dose of the VLP vaccine could be considered protective against pandemic influenza in women who become pregnant.

Although vaccination was performed prior to pregnancy, our data indicate that this VLP vaccine does not induce negative side effects in either women or their infants during pregnancy. The vaccine induced a specific antibody response that was observed 21 months after immunization (the last time point observed) and was transferred to the newborns, showing that the VLP vaccine induced a long-lasting antibody response. Although more studies must be conducted to determine if VLP vaccination is safe during pregnancy, these data could be useful as a precedent to support safety and immunogenicity studies of this vaccine platform during pregnancy.

There were limitations in this study. Firstly, the sample size was limited in the placebo and intervention arms, so the statistical power was insufficient to measure the differences in pregnancy complications and birth outcomes. A second limitation was the collection of pregnancy-specific information, since this was not part of the initial clinical trial. Therefore, the information was heterogeneous and the results inconclusive. The most important confounding factor was the exposure to the natural influenza A(H1N1)pdm09 virus infection. Volunteers did not present clinical symptoms suggesting an influenza infection; however, since they were residents of the Mexico City metropolitan area during the pandemic outbreak, they were likely to have been exposed to the virus. This study was importantly limited by the lack of design—the clinical trial was not designed to include pregnant women. Avoiding pregnancy during the study was requested in the written informed consent. Here, we have reported the observations that we could perform on the volunteers who gained the added risk of becoming pregnant as well as observations on their infants. Nonetheless, this study represents a unique opportunity to understand the effect of an experimental anti-influenza VLP vaccine administered prior to pregnancy. Notably, the trial was performed during the particular conditions of a pandemic outbreak at the epicenter of the emergency. Although this study was limited by its sample size, these results could suggest that it is safe to be vaccinated with the anti-influenza A(H1N1)pdm09 VLP experimental vaccine before pregnancy.

## Figures and Tables

**Table 1:** Characteristics of the study population (women).

	Placebo (*n* = 16)	VLP 15 μg (*n* = 23)	*p*	VLP 45 μg (*n* = 1)
**Age (mean ± SD)**	24.25 ± 2.43	27.44 ± 5.79	0.14 *	19
**Personal history of diseases**				
Denied, *n* (%)	16 (100%)	23 (100%)	1 **	1 (100%)
**Family history**				
Type 2 diabetes mellitus, *n* (%)	1 (6.25%)	4 (17.4%)		1 (100%)
Hypertension, *n* (%)				1 (100%)
Cancer, *n* (%)		1 (4.34%)		
Cardiovascular disease, *n* (%)				1 (100%)
Renal insufficiency, *n* (%)				1 (100%)
Hypothyroidism, *n* (%)				1 (100%)
Epilepsy, *n* (%)		1 (4.34%)		
**Time elapsed from vaccination to pregnancy**				
1 month, *n* (%)		6 (26%)		1 (100%)
2 months, *n* (%)		3 (13%)		
3 months, *n* (%)		3 (13%)		
4 months, *n* (%)		2 (8.6%)		
5 months, *n* (%)		2 (8.6%)		
6 months, *n* (%)		3 (13%)		
7–9 months, *n* (%)		2 (8.6%)		
>9 months, *n* (%)		2 (8.6%)		
**Breastfed the child**				
Yes, *n* (%)	4 (25%)	14 (60.8%)	0.049 **	1 (100%)
No, *n* (%)	12 (75%)	9 (39.2%)		

* Student’s *t* test comparing placebo vs. VLP 15 μg; ** Fisher’s exact test comparing placebo vs. VLP 15 μg. VLP—virus-like particle.

**Table 2:** Gestational age and delivery type in the placebo and 15 mg VLP vaccine groups.

Condition	Placebo, *n* (%)	VLP 15 μg, *n* (%)	*p* *
	16 (40)	23 (57.5)	
**Gestational age**			
Preterm	1 (5.5)	2 (8.7)	0.2
Term	15 (83.3)	20 (86.9)	0.8
Post-term	0	1 (4.3)	0.4
**Delivery type**			
Vaginal	8 (50)	11 (47.8)	0.9
Cesarean	8 (50)	12 (52.2)	0.9

* Fisher’s test.

**Table 3:** Obstetric complications in placebo and vaccinated women compared with the Mexican population.

Obstetric Complication	Placebo, *n* (%)	15 μg VLP, *n* (%)	*p* **	Reference Value *
	16 (40)	23 (57.5)		%
Preeclampsia	0	2 (8.6)	0.6	8
Fetal death	0	1 (4.3)	0.7	1.5
Premature rupture of membranes	0	1 (4.3)	0.7	5
Oligohydramnios	0	1 (4.3)	0.7	1.5
Gestational hypertension	0	1 (4.3)	0.7	10

* COMEGO: Clinic Gynecology and Obstetrics Guides in Mexico 2015, Mexico, Nieto Eds.; ** Fisher’s test comparing the placebo and the 15 μg VLP groups.

**Table 4:** Neonatal and one-year surveillance adverse events in infants of women in the vaccinated and placebo groups.

Condition	Placebo, *n* (%)	15 μg VLP, *n* (%)	*p* *
	16 (40)	23 (57.5)	
**Neonatal diagnosis**			
Low birth weight (<2500 g)	0	3 (13.0)	0.51
Fetal distress	0	2 (8.7)	0.6
**One-year surveillance period**			
Pneumonia	1 (6.25)	1 (4.3)	0.7
Hydrocele	0	1 (4.3)	0.1
Umbilical hernia	0	1 (4.3)	0.1
Gastroesophageal reflux	0	1 (4.3)	0.1

* Fisher’s test.
